# Fabrication of Silane-Grafted Cellulose Nanocrystals and Their Effects on the Structural, Thermal, Mechanical, and Hysteretic Behavior of Thermoplastic Polyurethane

**DOI:** 10.3390/ijms24055036

**Published:** 2023-03-06

**Authors:** Xuenan Sun, Xinze Yang, Jiajing Zhang, Bin Shang, Pei Lyu, Chunhua Zhang, Xin Liu, Liangjun Xia

**Affiliations:** 1 State Key Laboratory of New Textile Materials and Advanced Processing Technologies, Wuhan Textile University, Wuhan 430200, China; 2 College of Material Science and Engineering, Wuhan Textile University, Wuhan 430200, China; 3 Institute for Frontier Materials, Deakin University, Geelong, VIC 3216, Australia

**Keywords:** cellulose nanocrystal, thermoplastic polyurethane, surface modification, drawing, mechanical property

## Abstract

Reinforcement of polymer nanocomposites can be achieved by the selection of the appropriate fabrication method, surface modification, and orientation of the filler. Herein, we present a nonsolvent-induced phase separation method with ternary solvents to prepare thermoplastic polyurethane (TPU) composite films with excellent mechanical properties using 3-Glycidyloxypropyltrimethoxysilane-modified cellulose nanocrystals (GLCNCs). ATR-IR and SEM analyses of the GLCNCs confirmed that GL was successfully coated on the surface of the nanocrystals. The incorporation of GLCNCs in TPU resulted in the enhancement of the tensile strain and toughness of pure TPU owing to the enhanced interfacial interactions between them. The GLCNC–TPU composite film had tensile strain and toughness values of 1740.42% and 90.01 MJ/m^3^, respectively. Additionally, GLCNC–TPU exhibited a good elastic recovery rate. CNCs were readily aligned along the fiber axis after the spinning and drawing of the composites into fibers, which further improved the mechanical properties of the composites. The stress, strain, and toughness of the GLCNC–TPU composite fiber increased by 72.60%, 10.25%, and 103.61%, respectively, compared to those of the pure TPU film. This study demonstrates a facile and effective strategy for fabricating mechanically enhanced TPU composites.

## 1. Introduction

Cellulose nanocrystals (CNCs) have high strength, a high modulus, and a large specific surface area [1]. Furthermore, owing to their excellent mechanical properties, biocompatibility, physical properties, and chemical properties, CNCs are incorporated into polymers to nano-reinforce them [2,3,4,5]. However, CNCs contain a large number of hydroxyl groups with strong polarity; they thus tend to aggregate in hydrophobic polymers, consequently degrading the mechanical properties of CNC nanocomposites [6]. Therefore, improving the interfacial interactions between CNCs and the polymer matrix is key to fabricating reinforced polymer composites, which can be achieved by the surface modification of CNCs. Commonly used surface modification methods for CNCs include silanization, urethanization, amidation, and polymer grafting [7,8,9,10,11]. Many studies have indicated the importance of modifying CNCs for the enhancement of the interface interactions between the filler and matrix to improve the mechanical properties of the composites.

CNCs are a typical nanomaterial with a high aspect ratio; therefore, the mechanical properties of CNC–polymer composites can be further improved by orienting the CNCs in one direction using an external force. Commonly used methods include the application of a strong magnetic field, shear-solvent casting, and drawing [12,13,14,15,16]. The drawing method is considered an effective method for preparing high-mechanical fibers. For instance, CNCs were successfully aligned in polyurethane (PU) through melt-spinning to yield nanocomposite fibers with enhanced mechanical performance compared to films with randomly distributed CNCs [12]. CNC alignment was achieved by dry-jet wet spinning, and the as-prepared polyetherimide/CNC nanocomposite fibers exhibited significantly improved mechanical properties, with the tensile strength at a draft ratio of 7 being three to four times higher than that at a draft ratio of 2.8 [13]. Cellulose–chitosan composite fibers were fabricated by wet spinning. The drafting process occurred simultaneously as the primary fibers were formed. The strength of the composite fibers increased by 44.44% when the drafting ratio was 50:70 [14]. CNC/poly(vinyl alcohol) composite fibers were produced by dry-jet wet spinning, followed by wet-drawing. In this method, drawing is carried out after the formation of the primary fiber, but before drying. The wet-drawn process increases the stress of the composite fiber, but simultaneously decreases the strain of the fiber [15].

Additionally, the processing method and parameters of composite materials are also very important to the mechanical properties of composite materials. Wet spinning is a commonly used method for fabricating polyurethane composite fibers. However, the traditional wet spinning method using *N*,*N*-dimethylformamide as a single solvent in the short drawing process easily produces defects owing to its fast solidification speed [17]. Therefore, according to previous studies [17,18,19], to obtain composite materials with good mechanical properties, comprehensively considering the appropriate processing methods and corresponding parameters is necessary.

Herein, we present a nonsolvent-induced phase separation (NIPS) method with ternary solvents to prepare thermoplastic polyurethane (TPU) composites with excellent mechanical properties using 3-Glycidyloxypropyltrimethoxysilane-modified CNCs (GLCNCs). The mechanism of improving the mechanical properties of GLCNC–TPU composite films using ternary solvents and surface grafting of GL on CNCs was analyzed. Furthermore, we fabricated TPU composite fibers by wet spinning and drawing, and tested their mechanical properties. Our results showed that significant improvements in the mechanical properties, including stress, strain, and toughness, were achieved at very low GLCNC contents. This study focuses on understanding and gaining insight into the improvement of the mechanical properties of CNC composites.

## 2. Results and Discussion

### 2.1. GL Modification of CNCs

**Figure 1:**
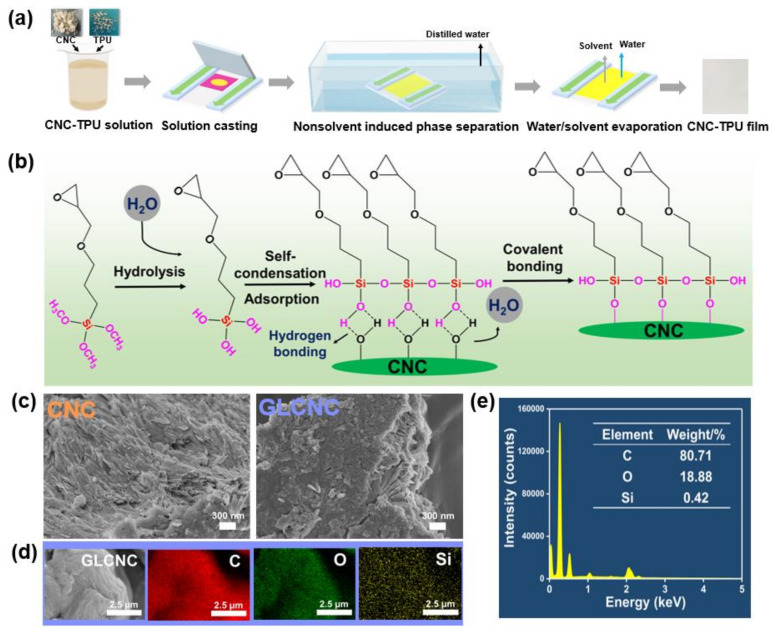
(**a**) Preparation process for CNC/TPU composite films; (**b**) surface modification of CNCs with GL; (**c**) SEM images of CNCs and GLCNCs; (**d**) SEM-EDS mapping images of GLCNCs; (**e**) and EDS spectroscopy of GLCNCs.

The surface modification of CNC with GL is depicted in Figure 1b. To demonstrate that the CNCs were successfully modified by GL, a SEM study was performed on the CNC and GLCNC. As shown in Figure 1c, the surface of the GLCNCs was rougher than that of the CNCs. Furthermore, the appearance of Si in the GLCNCs indicates that GL was successfully grafted onto the surface of the CNC (Figure 1d,e).

**Figure 2:**
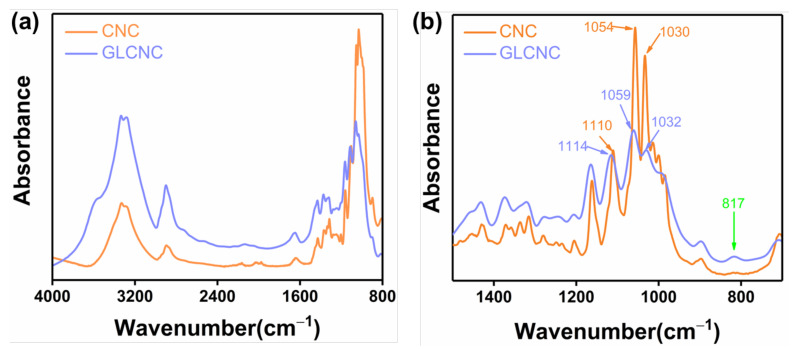
(**a**) ATR-IR spectra of CNCs and GLCNCs in the 4000–700 cm^−1^ range; and (**b**) deconvolution of ATR-IR spectra in the region between 1500 cm^−1^ and 700 cm^−1^.

Furthermore, the effect of GL on the microstructure of CNC was analyzed using ATR-IR and XRD. The ATR-IR spectra of CNCs and GLCNCs are shown in Figure 2. The ATR-IR spectrum of unmodified CNCs displayed characteristics corresponding to cellulose, with peaks at 3341, 2900, 1054, 1030, 999, and 1373 cm^−1^, which are assigned to the O–H, C–H, C–C, C–OH, and C–H stretching vibrations and the C–OH bending vibration, respectively [20,21]. The ATR-IR spectra in the 1500–700 cm^−1^ range were deconvoluted off-line using OMNIC 9.2, which tested the significance of individual hypothetical compounds in the presence of other unknown analytes (Figure 2b). After GL modification, the peak position is shifted from 1110 cm^−1^ to 1114 cm^−1^, which may be related to the stretching vibration of Si-O-C. Furthermore, the peak at 1054 cm^−1^ in the CNC spectrum underwent a shift in the GLCNC spectrum (1059 cm^−1^), which is ascribed to the large number of C–C bonds in GL [21]. Moreover, a new peak appeared in GLCNC at 817 cm^−1^, which corresponded with the stretching vibration Si-O-Si. These results demonstrated that chemical bonds have been formed after the CNC was modified by the GL.

**Figure 3:**
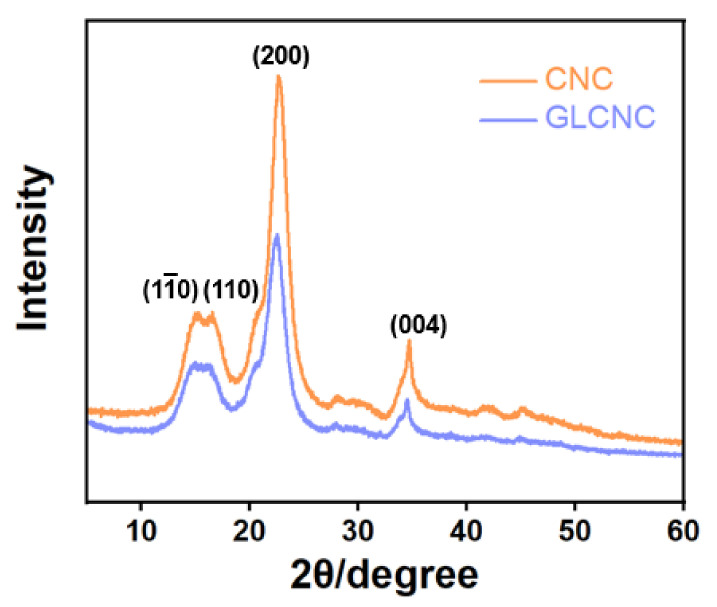
XRD patterns of CNCs and GLCNCs.

The crystal structures of the CNCs and GLCNCs were compared using XRD. The characteristic peaks at 14.9°, 16.4°, 22.8°, and 34.9° for unmodified CNCs correspond to their ($1\bar{1}0$), (110), (200), and (004) planes [22], respectively, indicating that they have a cellulose I-type crystal structure (Figure 3). In addition, the characteristic peaks of CNC were not shift upon treatment of GL, indicating that the cellulose I-type crystal structure of the GLCNCs was retained.

The crystallinity index (CRI) was calculated according to Segal et al. and is given by equation as follow [23]:

$$\mathrm{CRI}=\frac{I_{200}-I_{\mathrm{AM}}}{I_{200}}$$

where I_200_ is the height of the peak (200). I_AM_ is the minimum intensity at the valley between peak (200) and (110).

However, the crystallinity was reduced from 81.26% for the CNCs to 79.29% for the GLCNCs. These results confirmed that GL was successfully grafted onto the surface of CNCs without significantly damaging the crystal structure of the CNCs.

### 2.2. Structure of CNC/TPU Composite Films

**Figure 4:**
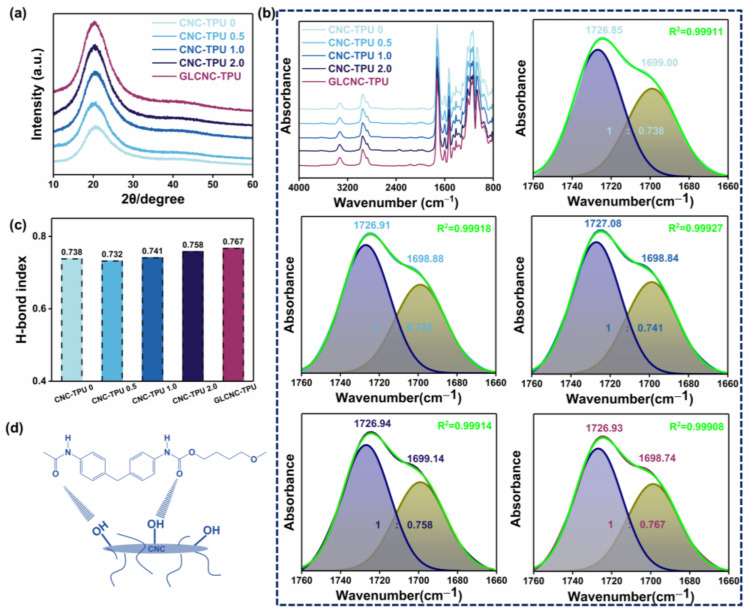
(**a**) XRD patterns of CNC/TPU and GLCNC-TPU composite films; (**b**) ATR-IR spectra of CNC/TPU and GLCNC-TPU composite films in the spectral regions 4000–800 cm^−1^ and 1760–1660 cm^−1^; (**c**) H-bond index of CNC/TPU and GLCNC-TPU composite films; and (**d**) schematic illustration of H-boding interactions between CNC and TPU.

The XRD patterns of the CNC/TPU and GLCNC–TPU composite films are shown in Figure 4a. The pattern of the CNC–TPU0 film displays a diffraction peak at 2θ = 20.6°, indicating the amorphous nature of the polymer. The XRD patterns of CNC/TPU and GLCNC-TPU composite films clearly display the peak assigned to TPU, indicating that the unmodified and GL-modified CNCs did not obviously affect the structure of the TPU. Additionally, the CNC characteristic peaks were absent for CNC/TPU and GLCNC-TPU composites because only a very small amount of CNC and GLCNC was added, and the surface of most CNCs may have been covered by TPU.

Figure 4b shows the ATR-IR spectra of CNC/TPU and GLCNC–TPU composite films, respectively. The CNC/TPU and GLCNC–TPU composite films retained all the main peaks of TPU at 3330, 2951, 1725, 1700, 1597, 1528, 1219, 1161, and 1069 cm^−1^, indicating that the addition of unmodified or modified CNCs did not significantly change the structure of the TPU. However, compared to the spectrum for CNC-TPU 0, no new peaks appeared for the CNC/TPU and GLCNC–TPU composite films. This may be because the characteristic bands were overshadowed by other bands.

Curve fitting of the carbonyl stretching region was performed to observe the changes in the structure of the composite films. The absorbance bands located at 1726 and 1703 cm^−1^ were ascribed to the splitting of carbonyl absorption, corresponding to the stretching vibration of free C=O and hydrogen-bonded C=O groups [24], respectively. The C=O groups could be curve-fitted by two Gaussian bands: free and hydrogen-bonded C=O. The carbonyl hydrogen-bond index (H-bond index) was calculated as the ratio of the peak area of H-bonded C=O to that of free C=O. As depicted in Figure 4c, with the increase in CNC, the H-bond index of the CNC–TPU composites first decreased and then increased. This reflects the existence of new H-bonds between the CNCs and TPU, which may have been between the free C=O in TPU chains and –OH on the surface of the CNCs (Figure 4d). In addition, the H-bond index of GLCNC–TPU was higher than that of CNC–TPU 1.0. This could be attributed to the modification of the CNCs, which limits the CNC aggregation and improves the interactions between the CNCs and TPU.

### 2.3. Mechanical Properties of CNC/TPU Composite Films

**Figure 5:**
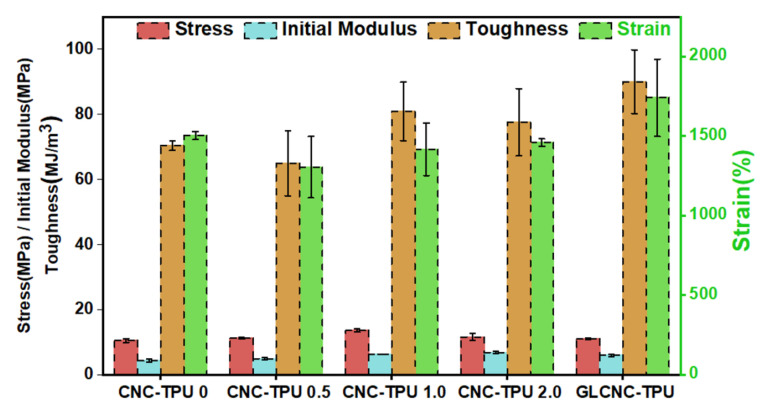
Stress, strain, initial modulus, and toughness of CNC/TPU and GLCNC-TPU composite films.

The stress, strain, initial modulus, and toughness are compared in Figure 5. Stress and initial modulus increased with the addition of CNCs. This is because of the increased H-bond index and addition of rigid CNCs. Compare to CNC–TPU 0, the stress of CNC–TPU 1.0 increased from 10.51 MPa to 13.56 MPa, initial modulus increased from 4.31 MPa to 6.20 MPa, and strain decreased slightly from 1502.74% to 1416.93%. The stress of the CNC-TPU 2.0 composite film decreased with the further increasing CNC content; this is because more CNC aggregated, resulting in the formation of stress concentration points, which is not conducive to stress transfer between the TPU matrix and CNCs.

The incorporation of GLCNCs had the greatest influence on the enhancement of tensile strain and toughness compared with those of CNC–TPU 1.0, with GLCNC–TPU exhibiting strain and toughness values of 1740.42% and 90.01 MJ/m^3^, respectively. The enhancement may be due to the following three factors. First, the dispersed GLCNCs acted as plasticizers between the TPU chains, increasing the free volume. Second, the increased H-bond value in GLCNC–TPU increased the interfacial interaction between TPU and the filler. Lastly, the slipping of GLCNCs, unwinding of TPU chains, and unwinding of the molecular chains of the CNC surface further improved the strain and toughness at break of the GLCNC-TPU composite film.

**Figure 6:**
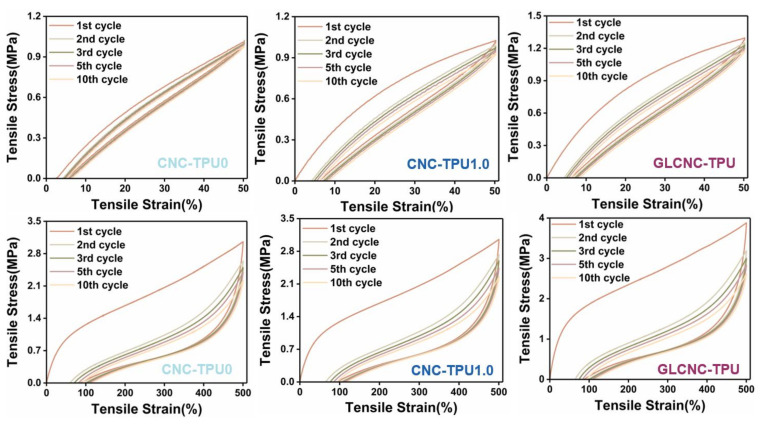
Stress–strain curves of CNC-TPU 0, CNC-TPU 1.0, and GLCNC-TPU composite films under cyclic loading at 50% and 500% strain.

The mechanical properties of TPU composites directly affect their practical application, particularly their resistance to repeated deflections. To assess the elasticity and damping behavior of the CNC/TPU composite films, the material response to cyclic loads was investigated. The typical loading–unloading curves of CNC/TPU and GLCNC–TPU composite films at strains of 50% and 500% are shown in Figure 6 and Figure A2. The loading–unloading behavior of all composites was nearly linear at 50% strain and highly nonlinear at 500% strain.

**Figure 7:**
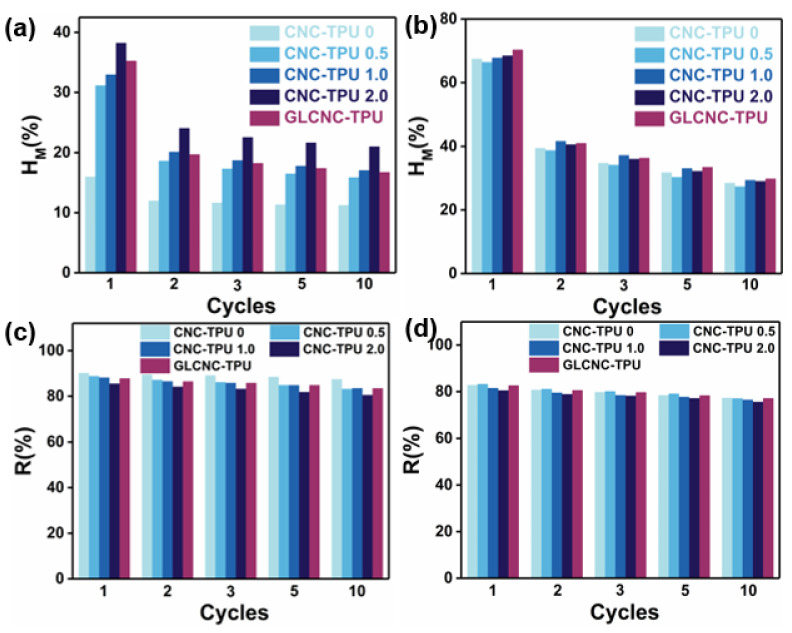
H_m_ of CNC/TPU and GLCNC-TPU composite films at (**a**) 50% strain and (**b**) 500% strain. R of CNC/TPU and GLCNC-TPU composite films at (**c**) 50% strain and (**d**) 500% strain.

For all composite films, the strain during unloading did not return to zero, indicating they underwent softening. The residual strain of CNC/TPU composite films at 50% and 500% strain increased with increasing CNC content and cycle number. However, the R of CNC/TPU composite films was still high after 50% and 500% cyclic stretching (Figure 7), demonstrating that the as-prepared composites exhibited good elasticity.

The calculated H_m_ and R values are shown in Figure 7, respectively. Typically, the H_m_ decreased during the first cycle and remained approximately constant in the subsequent cycles; the difference between cycle 2 and cycle 10 is minimal for all composites at 50% and 500% strains. The H_m_ of TPU–CNC 2.0 was 38.20% at 50% strain, which was greater than that of CNC–TPU 0 (15.97%) by 139.19%. Below the yield point, the behavior of CNC–TPU 0 resembled pure elasticity, whereas the CNC/TPU composite films were viscoelastic. However, the H_m_ of CNC–TPU 0 and CNC–TPU 2.0 were 67.83% and 68.42% at 500%, respectively, indicating a typical viscoelastic plastic. It was observed that H_m_ was relatively dependent on the CNC content at low strains. This was possibly caused by the more rigid and interconnected CNCs hindering the motion of the TPU chains. Under a large strain, the composites were weakened mainly by breaking hydrogen bonds, disentangling chains, and breaking aggregates [22,25,26].

As shown in Figure 6, the cycling stress–strain behavior of the GLCNC–TPU composite film was similar to that of the CNC–TPU 0. Meanwhile, the H_m_ exhibited by GLCNC–TPU was larger than that of CNC–TPU 1.0 in the first cycle, but was similar in subsequent cycles at 50% and 500% strains. By cycling the composite film under the same strain, GLCNC–TPU was transformed from a material that exhibited efficient energy dissipation to one that exhibited efficient energy recovery and storage. Additionally, the GLCNC–TPU exhibited a smaller plastic strain after each loading–unloading cycle at 500%, and a good elastic recovery of 88% and 83% at 50% and 500% strains, respectively.

This phenomenon has been observed in other studies and can be explained by the following reasons. When the composites are stretched, the hydrogen bonds between the TPU and CNC interface can rupture, and some TPU and GL chains will deform. Upon further straining, some entangled TPU and GL chains disentangle. Simultaneously, some TPU and GL molecular chains orient in the tensile direction. The deformation of the GLCNC–TPU composite film breaks down the rigid CNC aggregates, and the inter-particle friction between GLCNCs dissipates more energy. When the TPU and GL chains are further extended, some molecules detach from the surface of the GLCNCs, creating new bonds. At a high strain, the chemical bonds break until the material fails. During the unloading process, an ideally orientated elastic network was formed after the first cycle. In the subsequent tensile cycles, some hydrogen bonds, disentangled chains, and broken aggregates that accumulated during cyclic deformation underwent rearrangement, re-entanglement, and reaggregation, respectively, leading to softening of the composites [22,25,26].

### 2.4. Thermal Degradation Behavior of CNC/TPU Composite Films

**Figure 8:**
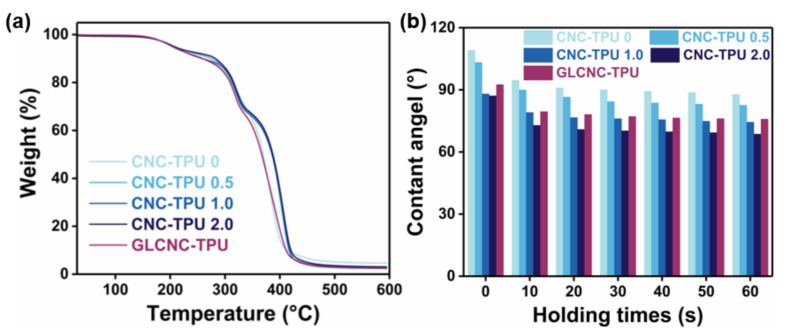
(**a**) TG curves and (**b**) water contact angle of CNC/TPU and GLCNC-TPU composite films.

The thermal stabilities of the CNC/TPU and GLCNC–TPU composite films were investigated using TGA. The thermal behavior of the composite films is shown in Figure 8a. The initial thermal decomposition temperature and main decomposition temperature were determined as the temperature at which 5% and 50% of the mass was lost, respectively [27].

As shown in Figure A3, CNCs underwent a loss in mass at approximately 100 °C, which can be attributed to the evaporation of absorbed water. The main decomposition of the CNCs occurred at approximately 303 °C, which is attributed to the breaking of cellulose chains. The initial thermal decomposition temperatures were almost the same after the introduction of different CNC contents into the TPU matrix. However, the main decomposition temperature was improved after the introduction of CNCs into the TPU matrix, suggesting that the thermal stability of the TPU film was improved by CNC incorporation. Compared to CNC–TPU 0, the main decomposition temperature of CNC–TPU 1.0. increased by 16 °C, which is attributed to the enhancement of interfacial interactions. In addition, the main decomposition temperature of GLCNC–TPU shifted slightly toward lower temperatures compared with that of CNC–TPU 0 owing to the low thermal stability of GL [28]. After incorporating GLCNCs into TPU, the composite presented almost similar thermal behavior to that of pure TPU, indicating that GLCNC–TPU composite film maintained good thermal stability.

### 2.5. Wettability of CNC/TPU Composite Films

The effects of CNCs and GLCNCs on the wettability of the CNC/TPU composite films are shown in Figure 8b. The water contact angle for the CNC–TPU 0 composite was found to be approximately 108°, indicating that it was hydrophobic. The hydrophilicity increased gradually with increasing CNC content, which was ascribed to the hydrophilicity of the CNCs and changes in the surface microstructure. A slightly higher water contact angle was obtained for the GLCNC–TPU composite than for CNC–TPU 1.0, indicating that the GLCNCs had little effect on the wettability of the composite film.

### 2.6. Morphologies of CNC/TPU Composite Films

**Figure 9:**
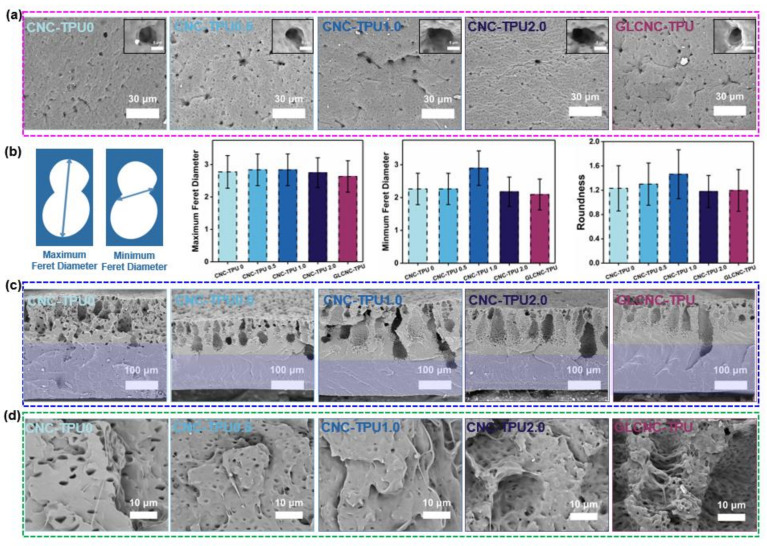
(**a**) SEM images of the surfaces of CNC/TPU and GLCNC-TPU composite films; (**b**) maximum Feret diameter, minimum Feret diameter, and roundness of pores on the surface of CNC/TPU and GLCNC-TPU composite films; (**c**) freeze-fractured and (**d**) tensile-fractured cross-section surfaces of CNC/TPU and GLCNC-TPU composite films.

To investigate the mechanism leading to the simultaneous enhancement in stress and toughness of TPU films upon the addition of GLCNCs, the freeze-fractured and tensile-fractured cross section surfaces of CNC–TPU 0, CNC–TPU 0.5, CNC–TPU 1.0, CNC–TPU 2.0, and GLCNC–TPU were examined, as shown in Figure 9. Similar to the TPU film prepared by the NIPS method with only DMF, the CNC–TPU 0 film also exhibited a porous cross-section and a surface featuring liquid–liquid phase separation. However, the macrovoids in the cross-section were significantly reduced, which is beneficial for improving the mechanical properties of the film. During the film-forming process, macrovoids are easily formed by the rapid convective flow of the non-solvent through the polymer solution. For CNC–TPU 0, TL is immiscible with water, and the velocity of convective flows decreases with the addition of other solvents, thus inhibiting the formation of macrovoids.

The surfaces of TPU and its composites displayed randomly arranged pores (Figure 9a). With increasing CNC content, the average Feret diameter and roundness of the pores first increased and then decreased (Figure 9b). When the CNC content was 2%, there were more lines around the pores, which may be related to the rigidity of the CNCs. Compared with CNC–TPU 1.0, the average Feret diameter of GLCNC–TPU decreased significantly, and the roundness value was close to 1.0, implying that GLCNCs have better compatibility than CNCs with TPU.

With an increase in CNC content, the roughness of the cross-section near the upper surface of the composite film increased (Figure 9c). Different ridges were observed in these regions. The temperature of liquid nitrogen is lower than the T_g_ of the soft segments, which makes the soft segments unable to immediately rearrange and presents the brittle fracture morphology of the cross-section. CNC–TPU1.0 and GLCNC–TPU exhibited mainly oblique and large ridges, while CNC–TPU2.0 exhibited small dense radial ridges. Additionally, GLCNC–TPU had the longest ridge, which may be due to the bridging effect of GL connecting the CNCs at different positions. Furthermore, the number of smaller pores in the region marked by the green box decreased, implying that the TPU was more compatible with GLCNCs than with unmodified CNCs. This better compatibility enabled stress to be efficiently transferred from the TPU to the GLCNCs, resulting in better mechanical properties of the composites.

Figure 9d shows the tensile fracture surfaces of different composites fractured at lab temperature. The SEM images of the tensile fracture surface of CNC–TPU 0 shows a porous and smooth surface, whereas those of the CNC/TPU composite films show irregular and relatively rough surfaces. This is attributed to the presence of CNCs and GLCNCs (white dots). When the CNC content was increased to 2%, the size of the white dots increased, indicating that they aggregated and formed defects, which led to a reduction in the mechanical properties. In addition, most of the CNCs in GLCNC–TPU were covered by the TPU with no obvious voids, as evidenced by Figure 9d (GLCNC-TPU). This may be due to two factors: first, after fracture, a layer of SS rearranged to cover the surface of TPU owing to the lower surface energy of SS; second, the rough surface of GLCNCs can enhance the interfacial adhesion between GLCNCs and TPU by providing “lock and key”-type mechanical bonding. Therefore, the load could be effectively transferred from the GLCNCs to TPU, which resulted in the improvement of mechanical properties of composites even with very low content of CNCs. Moreover, the entanglement between GLCNCs and TPU can slow down the propagation speed of cracks during tensile fracture. The high H-bond value requires consuming more energy during stretching, which contributes to the better toughness.

### 2.7. Mechanical Properties of GLCNC-TPU Composite Fiber

**Figure 10:**
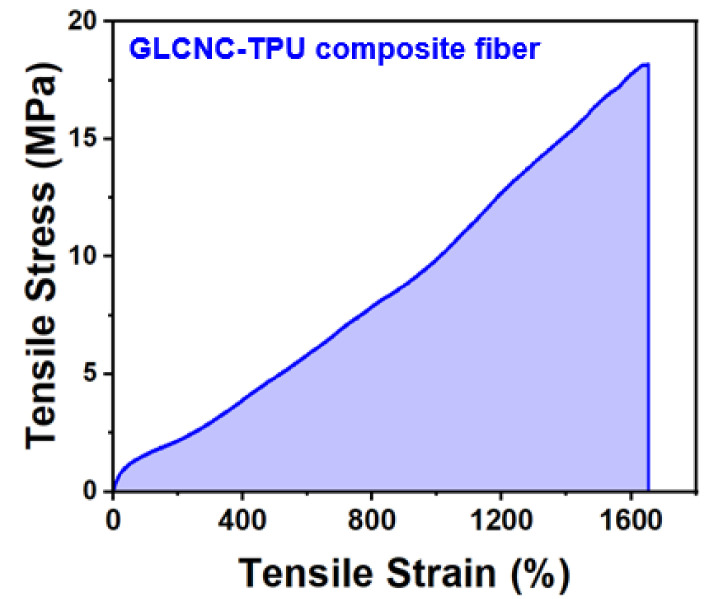
Stress–strain curve of GLCNC-TPU composite fiber.

The mechanical properties of CNC/TPU composites are strongly affected by the orientation of the CNCs; therefore, increasing the level of CNC alignment is an important parameter for improving the mechanical properties. We prepared a GLCNC–TPU composite fiber by wet-drawing after spinning for 10 min and drying under tension. The stress–strain curve of the GLCNC–TPU composite fiber is shown in Figure 10. The maximum stress, strain, and toughness achieved for the GLCNC–TPU composite fiber were 18.14 MPa, 1656.77%, and 143.20 MJ/m^3^, respectively. Compared to CNC–TPU 0, the GLCNC–TPU composite fiber showed a maximum increase of 72.60%, 10.25%, and 103.61% in stress, strain, and toughness, respectively. This implies that orientation leads to a better reinforcing potential for the GLCNC.

## 3. Materials and Methods

### 3.1. Materials

9370AU, a thermoplastic polyurethane (TPU) with a shore hardness of about 70, was obtained from Bayer Company. Cellulose nanocrystals (CNC) were provided by ScienceK. Co., Ltd. 3-Glycidyloxypropyltrimethoxysilane (GL) was purchased from Aladdin Biochemical Technology Co. Ltd. *N*,*N*-Dimethylformamide (DMF), toluene (TL), dimethylsulfoxide (DMSO), and ethanol were purchased from Sinopharm Chemical Reagent Co., Ltd. (Shanghai, China) All chemicals were used as received without further purification.

### 3.2. Synthesis of GL Modified CNC (GLCNC)

First, 250 mg CNC was dispersed in 30 mL DMSO by ultrasonication for 60 min. Then, 2 g GL was dissolved in 100 mL of distilled water and ethanol at 25 °C for 30 min and added to the CNC dispersion. Subsequently, the reaction was continued at 25 °C for 3 h by mechanical stirring. Finally, the mixture was washed 3–5 times, and the precipitate was collected by centrifugation and dried for 3 h to obtain GLCNC.

### 3.3. Fabrication of CNC/TPU Composite Films

The CNC dispersions (0, 0.5, 1.0, and 2.0 wt%) were prepared by dispersing CNCs in ternary solvents (DMSO:DMF:TL = 4:3:3 wt%) through an ultrasonicator at 100% power for 120 min. The CNC/TPU composite films were prepared though nonsolvent induced phase separation (NIPS) using ternary solvent as shown in Figure 1a. Firstly, TPU was dissolved in a certain amount of CNC dispersion at 30 °C for 120 min to obtain a CNC-TPU solution. Then, the as-prepared solution was poured into the glass mold and immediately transferred into a water coagulation at 30 °C for 120 min. Finally, the composite was dried to remove the water and solvents. The mass fraction of CNC in the dried composites was 0, 0.5, 1.0, and 2.0 wt%, and the composites were named as CNC-TPU 0, CNC-TPU 0.5, CNC-TPU 1.0, and CNC-TPU 2.0, respectively. Additionally, GL modified CNC in a composite with a content of 1.0 wt% was coded as GLCNC-TPU.

### 3.4. Fabrication of GLCNC-TPU Composite Fiber

The prepared spinning solution was GLCNC-TPU. The GLCNC-TPU composite fiber used a custom-designed fiber spinning unit through wet spinning. After the fibers were immersed in the water coagulation bath for 10 min, the fibers were stretched two times in the water coagulation bath and continued to be immersed in the water coagulation bath for 110 min to remove the residual solvents. Finally, the composite fiber was dried under conditions of tension to remove the water and solvents.

### 3.5. Characterization

**Figure A1:**
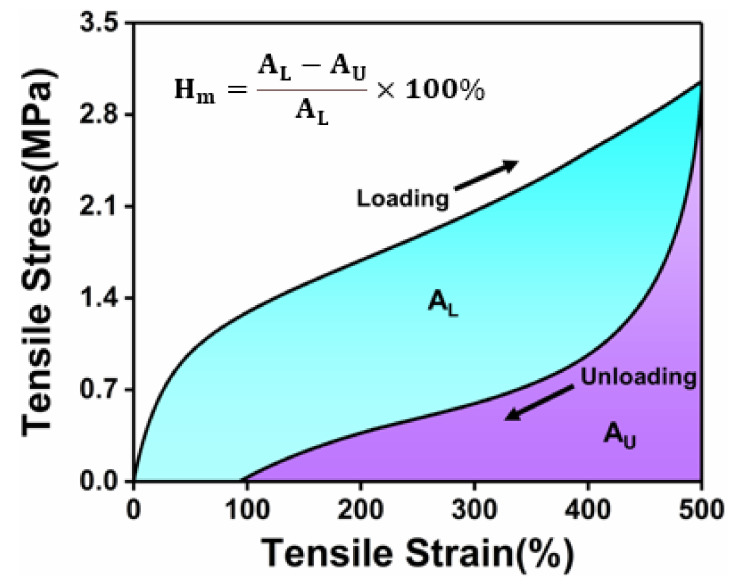
Schematic of the H_m_ quantification using the areas in the stress–strain curve.

**Figure A2:**
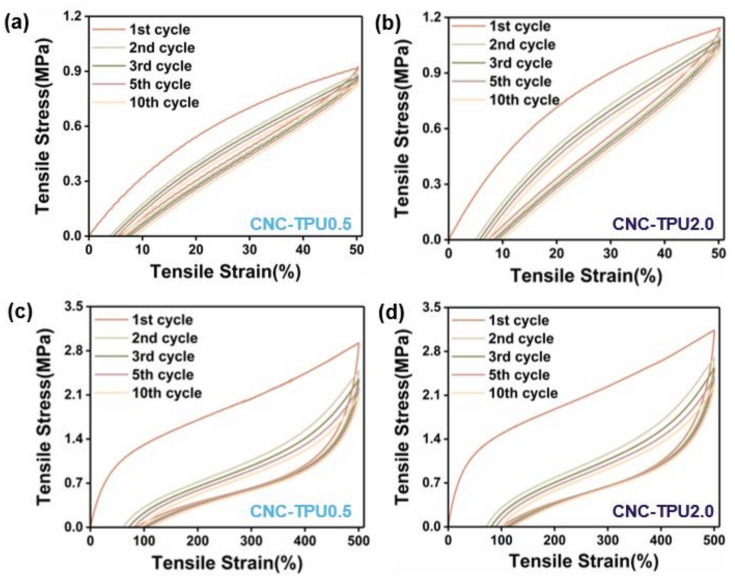
Stress–strain curves of CNC-TPU 0.5 and CNC-TPU 2.0 composite films under cyclic loading at 50% and 500% strain.

**Figure A3:**
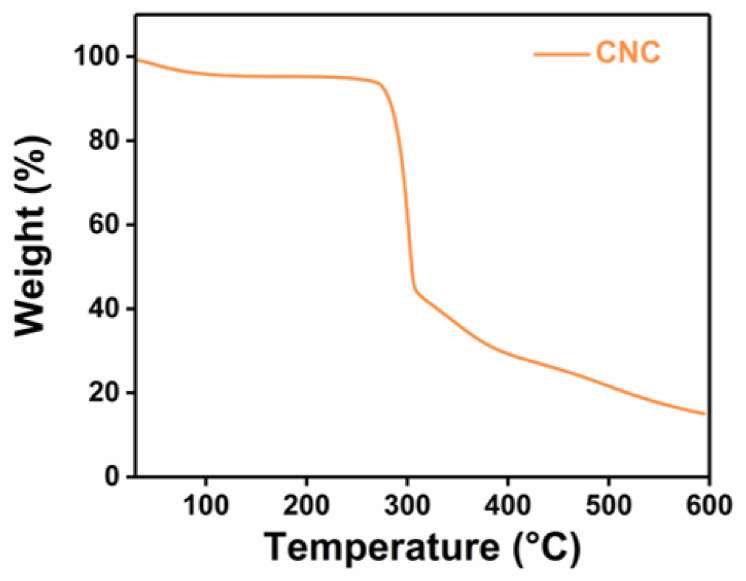
TG curve of CNC.

Attenuated total reflection infrared spectroscopy (ATR-IR) was conducted on a NICOLET IS-50 spectrometer equipped with an attenuated total reflectance attachment at the resolution 4 cm^−1^ and 128 scans. X-ray diffraction (XRD) was carried out on an Empryrean diffractometer, using Cu Kα at 40 kV and 40 mA in the range of 10–60°. The morphology and structure of CNC and CNC-TPU composites were observed with a scanning electron microscope (SEM, Phenom pure) and a field emission scanning electron microscope (FESEM, Zeiss sigma300) equipped with Energy Dispersive Spectroscopy (EDS). The mechanical properties of composites were tested by INSTRON 5943 at a crosshead speed of 100 mm/min. The results were averaged with at least five measured data. The cyclic tensile test was performed at 50 and 500% strain, 100 mm/min speed, and up to 10 cycles. The hysteresis (H_m_) for a given cycle is calculated by the ratio of the area bounded by the loading–unloading curves to the total area under the loading curve as shown in Figure A1. The elastic recovery (R) during cyclic loading–unloading is calculated as follows [29].

$$R=\frac{\varepsilon_{\max}-\varepsilon_{i}}{\varepsilon_{\max}}\times 100\%$$

where ε_max_ and ε_i_ are the maximum strain in the loading cycle and the strain at zero stress in the unloading cycle, respectively.

Thermogravimetric analysis (TGA) was carried out on a TG (TGA55) thermal gravimetric analyzer at a heating rate of 10 °C/min from room temperature to 600 °C under the nitrogen atmosphere. The wettability of the CNC-TPU composites was studied using contact angle measurement equipment (OCA15EC). Around 1 μL of deionized water was placed on the composites surface and the photograph of the water droplet was obtained.

## 4. Conclusions

In summary, TPU composites with excellent mechanical properties were prepared via NIPS with ternary solvents using GL-modified CNCs.

SEM-EDS, ATR-IR, and XRD analyses confirmed that GL was successfully grafted onto the surface of the CNCs without significantly damaging the crystal structure of the CNCs.

GLCNCs improve the interface interaction between TPU and GLCNCs. The addition of GLCNCs imparted the greatest increase in the tensile strain and toughness of the films compared with those of pure TPU, with strain and toughness values of 1740.42% and 90.01 MJ/m^3^, respectively. The GLCNC-TPU composite film exhibited a good elastic recovery rate and good thermal stability.

Furthermore, GLCNC-TPU composite fibers were prepared by wet spinning and subsequent drawing processes showing a maximum increase of 72.60%, 10.25%, and 103.61% in stress, strain, and toughness as compared with pure TPU. This study focuses on understanding and gaining insight into the improvement of mechanical properties.

## Data Availability

Not applicable.
